# Any Future for Faecal Microbiota Transplantation as a Novel Strategy for Gut Microbiota Modulation in Human and Veterinary Medicine?

**DOI:** 10.3390/life12050723

**Published:** 2022-05-12

**Authors:** Martina Takáčová, Alojz Bomba, Csilla Tóthová, Alena Micháľová, Hana Turňa

**Affiliations:** 1 Small Animal Clinic, University of Veterinary Medicine and Pharmacy, 041 81 Košice, Slovakia; ale.michalova@gmail.com (A.M.); hana.zborovska@uvlf.sk (H.T.); 2 Prebiotix s.r.o., 024 01 Kysucké Nové Mesto, Slovakia; alojz.bomba@iprobio.sk; 3 Clinic of Ruminants, University of Veterinary Medicine and Pharmacy, 041 81 Košice, Slovakia; csilla.tothova@uvlf.sk

**Keywords:** dogs, faecal microbiota transplantation, gut, probiotics, microbiome, modulation

## Abstract

Alterations in the composition of the intestinal microbiome, also known as dysbiosis, are the result of many factors such as diet, antibiotics, stress, diseases, etc. There are currently several ways to modulate intestinal microbiome such as dietary modulation, the use of antimicrobials, prebiotics, probiotics, postbiotics, and synbiotics. Faecal microbiota transplantation (FMT) represents one new method of gut microbiota modulation in humans with the aim of reconstructing the intestinal microbiome of the recipient. In human medicine, this form of bacteriotherapy is successfully used in cases of recurrent *Clostridium difficile* infection (CDI). FMT has been known in large animal medicine for several years. In small animal medicine, the use of FMT is not part of normal practice.

## 1. Introduction

Faecal microbiota transplantation describes a method of transfer of faeces from a healthy individual to the gut of a diseased recipient via an enema, endoscopy, nasogastric tube, or by indigestion peroral capsules [1,2,3]. The goal of therapy is to modulate and restore the intestinal composition of the recipient. At the present, the main indication for using this form of bacteriotherapy in humans is recurrent CDI unresponsive to antibiotic treatment [4]. There are other gastrointestinal and non-gastrointestinal diseases closely related to dysbiosis, in which the use of FMT has a beneficial effect. In large animal medicine, the therapeutic transmission of the rumen content known as transfaunation was described in the seventeenth century [5,6].

There are currently very few reports that describe the beneficial effects of FMT in acute and chronic diseases in small animals, so further research is required to bring this method into practice.

The main aim of this review is to summarize familiar knowledge about faecal transplantation in small animal medicine, as well as to cite similarities and differences with human medicine and to highlight its benefits, alternatives, and possible use in small animal gastroenterology in the future.

## 2. Gut Microbiome

The gastrointestinal tract of each individual is populated by a large number of bacteria, viruses, fungi, and protozoa. We refer to the community of all microorganisms in the digestive tract as the microbiota, while the intestinal microbiome is the organisms’ collective genome [7].

### 2.1. Microbial Diversity in Health

Bacteria have a dominant representation in all organisms that host the gastrointestinal tract. Their abundance increases from the stomach to the colon [8]. In a healthy canine stomach, the bacterial concentration ranges from 10^1^ to 10 ^6^ colony-forming units (CFU) per gram [9]. In the small intestine, the intestinal microbiota includes aerobic and facultative anaerobes, and the microbial concentration is approximately 10^2^ to 10^6^ CFU per gram. The colon is primarily occupied by anaerobes, with a bacterial density of approximately 10^11^ CFU per gram [9,10].

**Figure 1:**
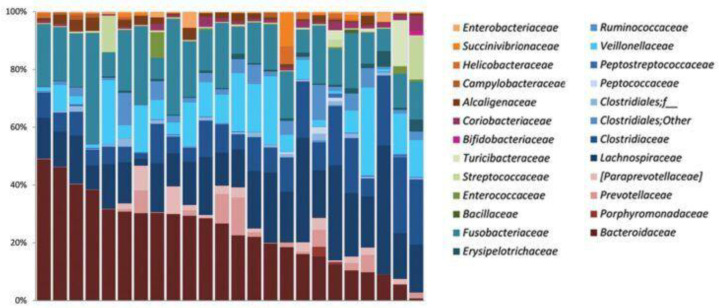
The diversity of the intestinal microbiota in healthy dogs [14,20]. Each column represents the composition of the microbiota in one healthy dog. Reprinted with permission from Suchodolski J [14].

However, every individual shows an individual microbial composition; the predominant phyla in the gastrointestinal tract (GIT) of healthy dogs and cats are *Firmicutes*, *Bacteriodetes*, *Fusobacteria*, and *Actinobacteria* [10,11]. Many significant species in this core bacterial community belong to the phylum *Firmicutes*. *Clostridia* is the most prevalent bacterial class, with three *Clostridium* clusters, IV, XI, and XIV, dominating. In addition to *Clostridia*, *Bacilli* and *Erysipelotrichi* are major classes within the phylum *Firmicutes* [12,13,14,15]. In dogs, the Fusobacteria phylum *Fusobacteria* is associated with a healthy canine microbiome, whereas in humans the presence of this phylum is associated with gastrointestinal diseases. This fact indicates that *Fusobacterium* has a different role in animals than in humans [12,13]. The abundance of Fusobacterium was found to increase in outdoor dogs and other carnivore species [12,16,17,18,19]. The diversity of the intestinal microbiota in healthy dogs is presented in Figure 1.

The human microbiota contains 10–100 trillion microbial cells harbored by each person. More than 1100 bacterial species and at least 160 species have been identified per individual [20,21]. The composition of the microbiome was found to depend on sex, race/ethnicity, age, diet, and the location of the gastrointestinal tract [20,21,22,23,24,25]. The dominant microbial phyla are *Furmicutes*, *Actinobacteria*, *Bacteriodetes*, *Proteobacteria*, *Fusobacteria*, and *Verrucomicrobia*; the two phyla *Firmicutes* and *Bacteriodetes* represent 90% of the gut microbiota [26]. The *Firmicutes* phylum consists of more than 200 different genera such as lactic acid bacteria (LAB), *Clostridium, Bacillus*, *Enterococcus*, and *Ruminococcus*.

*Bacteriodetes* include predominant genera such as *Bacteriodetes* and *Prvotella*. The phylum of *Actinobacteria* is less numerous and is predominantly represented by the genus *Bifidobacterium* [12,27].

### 2.2. The Function of the Gut Microbiome

The gut microbiome plays a large number of roles in the maintenance of health, but also in the pathogenesis of many diseases. Among all its functions, the most important are protecting the host against infectious agents, enhancing intestinal barrier function through tight junction formation, providing nutrients to the host, and modulating the immune system through cell-cell interaction (dendritic cells, Toll-like receptors) and through the production of microbial metabolites such as short-chain fatty acids (SCFAs), bile acids (Bas), tryptophan metabolites, and vitamins [28,29]. Some bacteria also produce antimicrobial substances that directly kill enteropathogens [30]. Due to the systemic transmission of these products and cells generated in the intestine, the positive effects of the gut microbiota can be detected both locally and in the surrounding organs. This phenomenon is referred to as the gut-organ axis, which includes the gut-brain, gut-skin, and gut-lung axes [31].

The intestinal microbiome, a separate organ, takes part in a variety of pathways [32]. These metabolic pathways of the intestinal microbiota and their effects on the host are described in Table 1. A balanced microbiome has a beneficial impact on host health. Imbalances in some of these pathways have a harmful effect. The most important pathways are BAs, SCFAs, and the indole pathway [32].

In dogs, *Clostridium hiranonis* is the main BA-converting bacterial species [33,34]. These bacteria convert BAs into secondary BAs in the dog colon (e.g., lithocholic and deoxycholic acids). In the colon, secondary Bas have several functions. They act as signalling molecules by binding to the natural receptors G protein-coupled bile acid receptor 1 (GPBAR-1), and they also maintain normal glucose concentration through the farnesoid X receptor [35]. In addition, they inhibit the germination of *Clostridium difficile* spores, whereas an increase in primary bile acids (an effect of dysbiosis) promotes the germination of bacterial spores [32]. A decrease in secondary BAs in the colon is observed in dogs with chronic enteropathies or after antibiotic treatment [34,36,37]. It is caused by a decrease in *C. hiranonis*, leading to an increased concentration of primary BA, the main etiology of secretory diarrhoea [32]. In such cases, FMT can reinstate *C. hiranonis*, leading to appropriate conversion from primary to secondary Bas [34].

Bacteria such as *Faecalibacterium*, *Turicibacter*, and *Ruminoccocus* ferment dietary carbohydrates to SCFAs (butyrate, acetate, propionate) [38]. These SCFAs represent a significant source of energy and growth factors for intestinal epithelial cells, act as nutrients that regulate intestinal motility, and create an unsuitable environment for pH-sensitive enteropathogens [39,40]. SCFAs also have immunomodulatory effects. For example, butyrate induces immunoregulatory T-cells, and acetate effectively modulates intestinal permeability [32].

Indole, a substance formed by metabolization of the amino acid tryptophane, improves intestinal permeability and increases mucin production [41]. Indole has also been shown to decrease the manifestation of interleukin 8, strengthen intestinal barrier function, and ameliorate enteropathy induced by nonsteroidal anti-inflammatory drugs in mice [42].

**Table 1:** The beneficial and harmful metabolic pathways of the gut microbiota and their impacts on the host.

		Consequence for Host		
Source	Bacterial Group Involved	Derived Metabolites	Beneficial	Harmful
**Dietary carbohydrates**	*Faecalibacterium*, *Bacteriodes, Ruminococcus, Blautia* [32].	Fermentation to SCFAs (acetate, butyrate, propionate) [38].	Anti-inflammatory effect. Maintenance of intestinal barrier function. Motility regulation. Source of energy for epithelial cells [38,39].	Virulence factors of enteropathogen activation (e.g., Salmonella type III secretion system) [32].
**Primary bile acids**	In small animals, mainly *C. hiranonis* [34].	Transformation to secondary BAs in colon [34].	Anti-inflammatory effect. Growth inhibition (*C. difficile, Clostridium perfringens, Escherichia coli*). Modulation of glucose/insulin secretion [35].	Secretory diarrhoea caused by lack of *C. hiranonis* (e.g., chronic enteropathies). In humans, a diet rich in fat, due to increased secondary BAs, represents a high risk of colon cancer [34,36,37].
**Dietary fat**	*C. perfringens, Bifidobacterium bifidum, Propiobacterium*) [32].	Conversion to hydroxystearic acids [32].	None [32].	Fatty acid diarrhoea [32].
**Dietary amino acid tryptophan**	Various [32].	Indole metabolites [43].	Anti-inflammatory effect. Maintenance of intestinal function [43].	Cytotoxic and putrefactive, but only in high concentrations. Indoxyl sulfate acts as a uremic toxin [32].
**Dietary amino acids tyrosine and phenylalanine**	Various [32].	P-cresol [32].	None [32].	Progression of chronic kidney disease similar to uremic toxin [32].
**Drug mycophenolate mofetil**	Various [32].	MPA (mycophenil acids) and acyl glucuronide [32].	None [32].	Production of proinflammatory cytokines causing diarrhoea [32].

## 3. Dysbiosis

Gut dysbiosis is defined as an imbalance in the structure of the gut microbiota that can result in functional alterations in the microbial proteome, transcriptome, or metabolome [44]. Dysbiosis is seen in a variety of pathologies, both systemically as well as locally, within the gastrointestinal tract [45]. Several factors impact the composition of the microbiota starting from the birth of an individual, including the type and quality of the diet the mother consumes, the composition of the maternal gut microbiota, stress, and the use of antibiotics [46]. In addition to these factors, there are several systemic or localized disorders that have an impact on the gut microbiome and are associated with dysbiosis [46]. Table 2 describes the most common conditions that lead to intestinal dysbiosis.

Individuals with intestinal dysbiosis show changes in the diversity of bacterial species, their amounts, and also their function, compared to healthy individuals [32]. Such changes in the microbiota lead to the destruction of the intestinal barrier, increasing the possibility for the translocation of pathogens and the development of disorders. The immune system can be activated, which in turn promotes inflammatory reactions. Other consequences of dysbiosis are changes in the concentration of bacterial metabolites [47]. This means that the dysbiotic microbiome may have negative consequences for the host. Possible consequences of the main types of dysbiosis are described in Table 3. There is also evidence that dysbiosis is associated with the occurrence of current conditions such as obesity, metabolic syndrome, or diabetes mellitus (DM) [48]. Changes in intestinal microbiota composition have been found not only in obese humans but also in animals with endocrine disorders [49,50,51,52,53]. Studies have shown that, in obese people, there is a shift in the ratio of *Firmicutes* to *Bacteriodetes,* as well as increased plasma concentrations of bacteria and their metabolites [54,55]. SCFAs (including butyrate) produced by *Clostridiales* strains (*Roseburia* and *Faecalibacterium prausnitzii*) were shown to decrease in people with type 2 diabetes, but non-butyrate producing *Clostridiales* were found to increase [56]. In addition to metabolic diseases, microbial imbalance is also associated with several other diseases, such as asthma and neurological dysfunction [57,58,59,60,61,62].

### 3.1. Dysbiosis in Canine Gastrointestinal Disorders

Gastrointestinal dysfunctions are the most evident association with intestinal dysbiosis. Most dogs and cats with gastrointestinal disorders have concurrent intestinal dysbiosis [47,64]. The gut microbiome tends to be altered during both acute and chronic conditions.

Acute gastrointestinal problems such as acute haemorrhagic diarrhoea (AHDS) and acute uncomplicated diarrhoea (AD) lead to strong alterations in canine microbial compositions with a decrease in SCFA-producing bacteria, such as *Firmicutes* and *Actinobacteria*, and increased abundance of *C. perfringen*, *E. coli*, and *Sutterella* [65,66]. *C. perfringens* is a commensal of the intestines and therefore can be identified in healthy individuals [67].

IBD (inflammatory bowel disease) is one of the most common chronic GIT diseases associated with intestinal dysbiosis. In this chronic condition, mucosa-adherent genera within the *Proteobacteria* (*E. coli*) have been found to increase, whereas *Bacteriodaceae, Prevotellaceae, Fusobacteria*, and *Clostridiales* have decreased [68]. In the study that described canine luminal dysbiosis in IBD, a decrease in the number of *Bacteriodetes* and Firmicutes was presented, and an increased abundance of *Actinobabacteria* and *Proteobacteria* was observed [69].

### 3.2. Dysbiosis Index

A novel approach, the dysbiosis index (DI) has been established to assess the canine faecal microbiota [42]. The qPCR assay quantifies the abundances of seven bacterial groups: *Faecalibacterium* spp., *E. coli*, *Turibacter* spp., *Fusobacterium* spp., *Streptococcus* spp., *Blautia* spp., and *C. hiranonis* [61], together with total bacterial count, and summarizes them in a single number (DI) [42]. A mathematical model of DI calculation has been described by AlShawaqfeh et al. [42]. The reference ranges of these bacterial groups are described in Table 4.

The DI should always be interpreted together with the abundance of the individual taxa. A DI below 0 represents a normal microbiota. A DI between 0 and 2 is equivocal, indicating a minor change in the microbiota. In such cases, the evaluation of follow-up samples might be performed a few weeks later. A DI > 2 points to microbiota dysbiosis. Most of these dogs have a decreased abundance of healthy *C. hiranoni* bacteria, as a result of the abnormal conversion of primary to secondary bile acids. The loss of secondary bile acids is a significant trigger for the development of dysbiosis in dogs [64].

**Figure 2:**
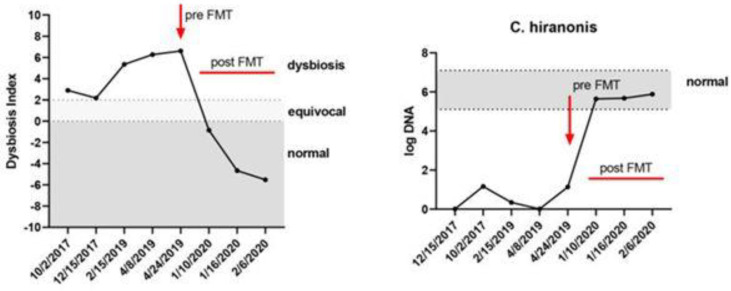
**A dog with persistent dysbiosis and recurrent *C. difficile* infection.** After FMT, the dysbiosis index normalized and the abundance of *C. hiranonis* increased. The dog was subsequently negative for *C. difficile* [32] From article under the CC BY-NC-ND license, no changes were made.

An increase in DI, together with a decrease in *C. hiranonis*, was noticed in dogs treated with antibiotics (metronidazole, tylosine), similar to dogs with EPI and chronic enteropathies [33,34,64], while dogs on proton-pump inhibitors (omeprazole) or raw food diets (BARF) have increased DI with a normal abundance of *C. hiranonis* [70,71]. In addition to determining normal versus abnormal microbiota, DI can be used to analyze changes in microbial composition over time or in response to treatment such as FMT (Figure 2).

## 4. Modulation of the Microbiome

While dysbiosis is a key factor in the pathogenesis of many gastrointestinal and systemic diseases, the recovery of the intestinal microbiota composition is a crucial therapeutic target. Currently, the intestinal microbiome can be modified by several possibilities such as diet, antimicrobials, prebiotics, probiotics, postbiotics, synbiotics, or FMT. Each of these forms has a different mechanism of action with beneficial effects and possible side effects [32]. Table 5 introduces the most common types of intestinal microbiota modulation in both dogs and humans.

### 4.1. Dietary Modulation

Dietary modulation should always be a part of gut microbiota modulation. The effect of diet modulation is based on the ability of a highly digestible diet to reduce the amount of undigested substrate in the intestinal lumen that leads to bacterial overgrowth. In addition to that, replacement of a diet with a novel or hydrolyzed protein results in a reduction in the inflammation response [72,73].

Sonnenburg et al. found that, in humans, a modern low-fiber diet leads to the loss of microbial diversity over generations [75,76,77]. Over the decades, people have changed their dietary habits. Gathered food was changed to farm food, later to mass consumption of processed food. Each dietary shift has led to changes in the microbiota [77,78]. These changes lead to an increasing rate of disorders such as IBD, IBS (irritable bowel syndrome), cardiovascular diseases, and metabolic disorders such as obesity, insulin resistance, type 2 diabetes, and nonalcoholic fatty liver disease (NAFLD) [77].

Dominika et al. mentioned that various components of the diet affect microbiota diversity. For example, whey consumption decreases the pathogenic bacteria *C. perfringens* and *Bacteroides fragilis*, while protein extracts of whey and pea lead to an increase in the commensal of lactic acid bacteria genus and *Bifidobacterium* [79,80].

Consumption of a high-saturated diet was found to increase the proportion of *F. prausnitzii,* while a low-fat diet was shown to increase the faecal abundance of *Bifidobacterium* [81].

Omega-3 polyunsaturated fatty acids (omega-3 PUFA), such as docosahexaenoic acid (DHA), eicosapentaenoic acid (EPA), alpha-linolenic acid (ALA), and docosapentaenoic acid (DPA), cannot be synthetized by the human body and therefore must be taken from the diet [82]. A diet rich in omega-3 PUFA, such as seafood, deep-sea fish (salmon, mackerel, sardines), nuts, and seeds, has several beneficial effects on the gut microbiota [83]. Consumption of such a diet leads to a decrease in the growth of *Enterobacteria* and an increase in the growth of *Bifidobacteria* [84,85]. Furthermore, the consumption of omega-3 PUFA leads to an increased production of anti-inflammatory mediators and the inhibition of pro-inflammatory mediators, which has a positive effect on microbiome modulation [82]. The positive effect of omega-3 PUFA is also due to the increased production of SCFAs such as butyrate, an essential source of energy for colonocytes [86]. It should not be forgotten that an inappropriate ratio of omega-3 to omega-6 results in an increase in the ratio of *Firmicutes* to *Bacteriodetes*, leading to the development of obesity and NAFLD [81].

### 4.2. Prebiotics

Prebiotics are defined as “non-digestible foodʺ ingredients (dietary fibers or carbohydrates) that beneficially affect the host by selectively stimulating the growth and/or activity of one or a limited number of bacterial species [42,87].

We can divide prebiotics into fermentable and nonfermentable. The first group consists of those prebiotics that can be fermented by colonic bacteria into SCFAs, with a variety of health benefits [39,88]. Fermentable prebiotics, including psyllium, pectin, guar, and fructo-oligosaccharides, also promote the growth of specific bacteria (e.g., *Lactobacilli* and *Bifidobacteria*) [39,87]. Psyllium, a soluble and fermentable dietary fiber, also contributes to the metabolism of BAs by binding BAs to the intestinal lumen [88]. The dose for dogs ranges from 0.5 to 1 g/kg of body weight [32].

### 4.3. Probiotics

The World Health Organization defines probiotics as “live microorganisms, which, when administered in adequate amounts, confer a health benefit on the host” [89,90]. They can be regulated as food supplements, medical food, or drugs.

There are several important mechanisms underlying the antagonistic effects of probiotics on various microorganisms that include the following:

- Enhancement of the epithelial barrier;
- Increased adhesion to intestinal mucosa;
- The concomitant inhibition of pathogen adhesion;
- Competitive exclusion of pathogenic microorganisms;
- Production of anti-microorganism substances such as organic acids, defensins [41,61,62], or specific toxins aimed at pathogens [66];
- Modulation of the immune system [91].

In humans, probiotics refer mainly to the genera lactic acid bacteria, *Bifidobacterium*, and include many different strains such as *Limosilactobacillus fermentum*, *Lactobacillus acidophillus, Lactobacillus johnsonii*, *Limosilactobacillus reuteri*, *Lacticaseibacillus paracasei*, *Lactiplantibacillus plantarum*, *Lacticaseibacillus rhamnosus*, *Bifidobacterium bifidum*, *Bifidobacteriumlongum*, *Bifidobacterium breve*, and *Bifidobacterium animalis* [79,92]. Mixtures of these strains are becoming increasingly popular as researchers gain a deeper understanding of increasing efficacy through possible additive or synergistic effects [93].

Studies on human and animal models show the clinical potential of probiotics against many diseases [94]. Probiotics have been reported to suppress diarrhoea [89,95], alleviate lactose intolerance [96] and postoperative complications [97], exhibit antimicrobial [98] and anti-colorectal cancer activities [99,100], reduce irritable bowel symptoms [101], and prevent inflammatory bowel disease [89,102]. In addition, *Bifidobacteria* and *Lactobacilli* have been successfully used for the prophylactic prevention of traveller’s diarrhoea [103]. Probiotics have shown good results in reducing inflammation, as well as regulating innate immunity and the corresponding signalling pathways [94].

In small animal practice, probiotics are used in cases of acute and chronic diseases of the gastrointestinal system. A study reports significant clinical improvement and decreased mortality in dogs with parvoviral enteritis in which a commercially available LAB (lactic acid bacteria) mixture “de Simone” formulation (*Lacticaseibacillus casei*, *Lactiplantibacillus plantarum*, *L. acidophilus*, *Lactobacillus delbrueckii*. subsp. *bulgaricus*, *B. longum*, *B. breve*, *B. infantis*, *Streptococcus salivarius* spp. *thermophiles* spp. *thermophilus*) is added orally to standard treatment compared to standard treatment alone [104,105]. In the acute haemorrhagic diarrhoea syndrome associated with *C. perfringens* overgrowth, the “de Simone” LAB probiotic mixture reduced clinical severity and increased the faecal abundance of intestinal bacterial markers (e.g., *Faecalibacterium* sp.), while *C. perfringens* were reduced [106]. In dogs with acute idiopathic gastroenteritis, a shorter duration of diarrhoea has been reported, as well as a better faecal score after 1–3 weeks of treatment after oral application of *B. animalis*.

The benefits of probiotics or synbiotics have also been investigated in chronic conditions of GIT in dogs and cats. In one study, the “de Simone” LAB mixture was administered to dogs with IBD (refractory to dietary and antibiotic treatment). This study confirmed that probiotic treatment was not inferior to the standard treatment, consisting of a combination of metronidazole and prednisolone in reducing clinical signs and inflammatory cells in duodenal bioptates [107,108,109,110].

In cats, probiotics have been used in the case of chronic *Tritrichomonas fetus* and chronic constipation [107,111,112]. In the case of *Tritrichomonas* infection, no clinical improvement was observed, but it significantly reduced relapses [107]. There is also evidence that the use of LAB probiotics in chronic feline constipation and idiopathic megacolon leads to clinical improvement [111].

Currently, there are more and more reports that question the effectiveness and safety of probiotics, mainly in high-risk patients. Because of that, there is increasing interest in a novel group of preparations: postbiotics [113,114,115].

### 4.4. Postbiotics

Postbiotics is a relatively new term that has been created to refer to the nonviable metabolic products of probiotics that act on the biological activity in the host. Some researchers believe that postbiotics are responsible for many of the beneficial effects of probiotics [116]. According to Tsilingiri et al., postbiotics include any substance produced through the metabolic activity of the microorganism that benefits the host directly or indirectly [117]. Although postbiotics do not include live microorganisms, they show beneficial properties through pathways similar to those seen in probiotics but with a lower risk of side effects. Currently, available classes of postbiotics include many different constituents, including metabolites, SCFAs, microbial cell fractions, functional proteins, extracellular polysaccharides (EPS), cell lysates, teichoic acid, muropeptides derived from peptidoglycans, and pili-type structures [46].

The mechanism of action of postbiotics is based on the pleiotropic effect, including anti-inflammatory, antioxidant, immunomodulatory, and anticancer properties [30]. Because of these qualities, postbiotics can be used in the treatment or prophylaxis of many disease units, including those for which effective treatment has not yet been found (e.g., IBD, Alzheimer’s disease, or multiple sclerosis) [117].

Currently, the use of postbiotics in the prevention and treatment of SARS-CoV-2 infection is mentioned, as the structure and metabolic activity of the intestinal microbiome may be related to the occurrence of biomarkers that predict the course of severe coronavirus disease (COVID-19) [118].

### 4.5. Antibiotics

The use of antibiotics (e.g., metronidazole, tylosin) in chronic GI diseases leads to the suppression of clinical symptoms. Relapse after finishing treatment can be explained by the fact that antibiotics reduce the bacterial load while improving clinical signs [119]. After a course of antibiotics, the bacteria regrow, which leads to a relapse in clinical signs [76,120]. Stopping antibiotic treatment also causes changes in the composition of the microbiota that last for months. In dogs, the use of metronidazole leads to an increase in *E. coli* and the reduction of beneficial bacteria, while the use of amoxicillin-clavulanic acid causes a decrease in the diversity of microbial species in cats [34,64,121]. Due to these negative side effects, antibiotic treatment should be recommended only in chronic cases, when anti-inflammatory and dietary trials have failed [32].

As antimicrobial resistance is becoming an increasingly common problem in human medicine, in veterinary medicine, there has been a rising trend to promote antibiotic usage that is appropriate and careful [122,123,124]. Antibiotics used orally may result in the development of resistant strains in the GIT, as well as cross-resistance to other antimicrobial drugs [125]. According to Gongora et al., oral treatment with amoxicillin or amoxicillin/clavulanic acid results in an increase in the proportion of ampicillin-resistant *E. coli* during treatment and an increased occurrence and proportion of ampicillin-resistant *Enterococci* during and after treatment [126]. As a result of growing antimicrobial resistance, the use of metronidazole to treat protozoal giardiasis infection is no longer as effective as it used to be [127,128]. Due to these negative side effects, antibiotic treatment should be recommended only in chronic cases when anti-inflammatory and dietary trials have failed [32].

## 5. FMT

One of the novel methods of modulating the gut microbiota is ”Faecal microbiota transplantation”. FMT means the administration of a faecal matter solution from a donor into the intestinal tract or recipient mainly to change the recipient’s microbial composition [129,130]. This procedure can be performed by duodenoscopy, nasogastric/nasojejunal tube, colonoscopy, enema, or by indigestion of peroral capsules [1,2].

### 5.1. History

The transfer of gastrointestinal matter is not a new method in veterinary medicine. In the animal kingdom, the consumption of faeces, called coprophagy, is observed in many species [131,132,133]. Thanks to this process, the gastrointestinal tract is developed, resistance to colonization of pathogens increases, and absorption of nutrition is improved [4]. The therapeutic transfer of rumen content (transfaunation) was described in Europe in the seventeenth century [5,6]. The indication for this therapeutic trial was ruminal acidosis in cattle and sheep and chronic diarrhoea in horses. It was also used to increase the resistance of newborn chicks to enteric pathogens [134,135,136].

In humans, the FMT method has been known in China since the fourth century CE [3]. Chinese medicine includes various forms of FMT, including fresh, dried, fermented, and infant-derived products that have been used for many gastrointestinal disorders [64]. In Europe, the German physician Franz Christian Paullini observed that, since manure had been used as fertilizer, faecal consumption has been common in humans and animals. In 1696, he also published the book *Hailsame Dreck Apotheke (Salutary Filth-Pharmacy)*, in which he described the medical uses of human and animal faeces [137].

In 1958, the team of Ben Eiseman provided a report describing the successful treatment of four patients with pseudomembranous colitis caused by *C. difficile* using faecal enemas. In this study, this condition was due to the use of antibiotics, leading to the suppression of the native microbial population that provides protection against pathogens [138]. They expected the procedure to be standardized and tested in clinical trials. However, the effectiveness of vancomycin for the treatment of pseudomembranous colitis was soon confirmed [138,139].

In human medicine, there is no doubt about the beneficial effect in patients with CDI, but what do we know about its effects and potential use in veterinary medicine?

### 5.2. Mechanism of Action of FMT

The mechanism of action in the intestinal microbiota has not yet been clearly identified. The crucial benefits of FMT in patients with CDI include an increase in bacterial species diversity and a change in the microbial profiles toward those of healthy donors [137,140].

Patients with CDI are known to have gut dysbiosis characterized by higher levels of *Proteobacteria* species and lower levels of *Firmicutes* and *Bacteriodetes* species. The administration of FMT may lead to *Firmicutes* and *Bacteriodetes* communities and decrease *Proteobacteria* [141].

In addition to creating less favourable conditions for the growth of *C. difficile* by providing bacteriocines, the administration of faecal matter triggers a mechanism known as competitive exclusion of pathogens [137]. This mechanism includes the restoration of the prevalence of secondary bile acids over primary bile acids in faeces [137,140]. Primary bile acids have been shown to stimulate spore germination, while secondary bile acids are potent inhibitors of spore germination [20]. A high concentration of secondary bile acids leads to the outcompeting of *C difficile* for nutrients and an unfavourable environment for its growth [137]. It is worth mentioning that the modulation of the intestinal microbiota by transplanted faeces leads to an increase in the utilization of sialic acid by commensal bacteria. This utilization results in the deficiency of the carbohydrate energy source for *C. difficile* [142].

By secretions of mucin, the transplanted faecal material contributes to reestablishing the integrity of the intestinal barrier [140]. Furthermore, the administration of faecal matter is beneficial for modulating the mucosal immune response and reducing the inflammatory response due to the production of butyrate-producing species of bacteria [140,143,144]. It is also likely that the favourable effects are supported by bacteriophages found in the donor’s faeces [144].

### 5.3. Forms of Application

There are several routes available for FMT administration, such as colonoscopy, naso-gastric duodenal, jejunal infusion, enema, or oral capsule ingestion [145,146,147]. Each of these methods has some limitations, for example the risk of vomiting and aspiration pneumonia during the administration of the naso-gastric tube, difficulties in retaining administered suspension for enema, or the risk of tissue perforation for jejunal infusion and colonoscopy [148].

Oral capsules were introduced to address limitations and gaps that had previously been noticed in other FMT delivery methods. They are noninvasive, the cheapest, and the easiest mode of administration to store. Using this form of FMT eliminates several procedural risks encountered in other FMT treatment routes. Kao et al. showed that oral capsules are an effective approach in the treatment of rCDI (refractory *Clostridioides difficile* infection) as colonoscopy [2]. Administration of these capsules is associated with side effects, including vomiting, aspiration, and failure in reaching their intestinal target [147,149].

FMT can be divided into two groups. The first is autologous transplantation using the patient’s own faeces, which are collected prior to any treatment. This form of faeces transfer is successfully used to restore the composition of the destroyed microbiota by antibiotic use during allogeneic hematopoietic stem cell transplantation [149,150]. The second group includes allogenic FMT involving the use of a related or unrelated healthy donor’s faecal sample [151]. Allogenic transplantation has appeared to be very effective in the case of rCDI [152].

### 5.4. Recommendations for the Use of Faecal Microbiota Transplantation in Humans and Dogs

#### 5.4.1. Donor Selection in Humans

With the increasing success of FMT in the treatment of various diseases, there is a growing demand to standardize the preparation of faecal material, using accepted standards for delivery, ensuring safety for the donor and recipient.

In human medicine, faecal donors are healthy volunteers subjected to very strict screening procedures to avoid the spread of infectious or other diseases and to ensure the transplantation of a desirable faecal microbiome and metabolome [153]. Donor stool is provided from two sources: patient-directed donors and universal donors through stool banks [154,155]. Patient-directed sources are identified by the recipients and include family member or friends. The use of universal donors has emerged as the most used method in the United States of America. Universal donors include healthy volunteers who are young and have a normal body mass index, who are able to provide stool after examination [148].

The preliminary interview and laboratory tests are two crucial phases in the screening of potential donors [156]. The first set of questions looks at a variety of risk variables in order to reduce the probability of transmitting infectious pathogens or adverse bacterial species [157]. Potential donors are asked about their diet habits, the history of recent drug use (antimicrobials, corticosteroids, proton-pump inhibitors, chemotherapy drugs) and the history of diseases (diabetes, cancer, obesity, allergies, gastrointestinal, autoimmune, cardiovascular, or psychiatric disorders) [157,158]. The optimal donor corresponds to a young individual (preferably less than 50 years).

Donors with a permissive medical history must undergo blood and faecal examinations to rule out infectious diseases that can be transmitted by faeces [156]. Each candidate is subjected to the analysis of a complete blood count, including a panel of haematology and biochemistry, serology for infectious diseases such as hepatitis virus and human immunodeficiency virus [1,157]. Stool-testing includes common enteric pathogens such as *C. difficile*, faecal parasites, and *Helicobacter pylori* antigen (this last exam only for the upper route of FMT) [157].

Due to the emergence of the COVID-19 pandemic, a group of worldwide experts has proposed incorporating the SARS-CoV-2 test using nasopharyngeal swabs or stool RNA detection [159]. Finally, the candidate is accepted as a stool donor if the blood and faecal tests are negative [157].

#### 5.4.2. Donor Selection in Dogs

In canine medicine, there are not many studies on screening protocols for canine donors. Chaitman and Gaschen introduced general screening criteria to ensure that the faeces used for FMT are safe and of optimal quality for the recipient [153]. These selection criteria are summarized in Table 6.

#### 5.4.3. Preparation and Administration of the Faecal Solution

In human medicine, it is recommended to use a fresh stool sample within six hours after defecation. The faeces should be kept at room temperature during this period. To protect anaerobic microorganisms, preparation should be conducted as soon as possible. The faecal material (50 g) is mixed with a sterile saline solution (0.9%) (250 mL) by using a blender or manual effort, then homogenized and filtrated to avoid infusion syringe clogging [160]. In some studies, water has been used successfully as a solvent for faecal material [161,162,163]. According to studies, an FMT performed with fresh and frozen faecal samples has a similar efficiency for the treatment of rCDI [163,164].

Before freezing, glycerol is added as cryoprotectant to the final concentration of 10%. The final faecal solution should be stored at −80 °C. Various volumes (~300–700 mL) of faecal infusions have been used for individual transplants in people. On the day of the faecal infusion, the faecal suspension should be thawed in a warm (37 °C) water bath. After thawing, a saline solution could be added to obtain the desired suspension volume. The thawed faecal material should be infused within 6 h after defrosting [165].

In human studies, capsules containing donor faeces have been successfully used to deliver the desired microbiota per person. This could possibly serve as an alternative route of administration for veterinarians [160].

In veterinary medicine, there are very similar protocols used to prepare a faecal solution. Twenty to one-hundred grams of donor faeces, within 6–12 h after defecation, are typically used for the FMT procedure. Then, 1 volume of faeces is mixed with 4 volumes of 0.9% NaCl and filtered. Then, glycerol is added to the final concentration of 10% and stored at −80 °C [1,2].

### 5.5. Recent Uses of FMT in Small Animals

In common, there are not many reports describing the effect of FMT in small animal medicine [153]. Burton et al. tried to prevent postweaning diarrhoea in puppies by oral administration of the faecal inoculum. In this case, no clinical improvement was noticed [166]. In another study, the researchers tried to increase the survival of puppies with parvovirus infection by a combination of standard treatment with FMT. This trial did not markedly improve survival, but the hospitalization time was shorter, and the resolution of diarrhoea was reduced to two days [167].

Chaitman et al. performed a trial comparing the 7-day oral application of metronidazole (15 mg/kg q 12 h) with an administration of faecal material via enema in dogs with uncomplicated non-infectious diarrhoea. Although the consistency of the faeces improved after one week of cure in both groups, only in patients treated with FMT, the firmer faeces were seen on day 28. The faecal dysbiosis index did not improve in most patients receiving metronidazole, while it normalized after one week in most dogs treated with FMT [34,42].

An increase in faecal bacterial diversity of beneficial bacteria such as *C. hiranonis* and *Faecalibacterium* and a decrease of *E. coli* was noticed only in dogs treated with FMT [64].

A case report with a toy poodle suffering from refractory IBD confirmed the positive effect of FMT even in the case of chronic diseases. This dog received nine FMTs via enema. After a 6-month period, the dog’s Clinical IBD Activity Index and faecal consistency improved [168].

Another study described an 8-month-old French bulldog with chronic colitis and positive *C difficile* faecal culture. This dog received a single oral dose of FMT. The frequency of defecation and the consistency of faeces improved significantly after 2 to 3 days. Relapse was not observed for at least 6 months [167].

According to Chaitman et al., the administration of a single FMT in dogs with acute diarrhoea (AD) seemed to be very successful [34]. The use of an FMT instead of antibiotics leads to the prevention of negative consequences such as lower microbial diversity, changes in specific bacterial taxa, abundance, and metabolic shift [34,153]. An FMT also shows promising results as a treatment for dogs with chronic diseases such as chronic enteropathies or exocrine pancreatic insufficiency. Unfortunately, improvement after a few days of applying a faecal transplant is often followed by relapses. Therefore, numerous FMTs can be required in most situations [34].

Nowadays, in small animal practice, an FMT has the potential to improve health in acute and chronic diseases associated with dysbiosis. As there are a few studies on its use, its standardization requires further research.

### 5.6. Experience with Faecal Microbiota Transplantation in People

In human medicine, faecal microbiota transplantation represents the most effective means to therapeutically manipulate the gastrointestinal microbiome. This technique is now recognized as the treatment of choice for life-threatening recurrent *Clostridioides difficile* infection (rCDI) [2,5]. *C. difficile*, Gram-positive, anaerobic, spore-forming, and toxin-producing bacillus, leading to nosocomial infection, causing clinical signs ranging from mild watery diarrhoea to a severe condition called pseudomembranous colitis [169,170]. FMT gained widespread acceptance during the CDI epidemic, where it achieved resolution rates approaching 100%. During the height of the epidemic, CDI was responsible for approximately 30,000 deaths [162]. In these hard-to-treat cases, the consistency of FMT achieved cure rates of >90% [171].

Although *C. difficile* is native to the distal intestine, its growth and pathogenic activity are normally inhibited by the commensal microbiota [4]. Infection occurs more frequently when patients receive antibiotics that alter their normal enteric gut bacteria, allowing the overgrowth of *C. difficile* [172]. In patients with rCDI, bacterial diversity is markedly decreased. The abundance of *Firmicutes* and *Bacteriodetes* is reduced, whereas members of the *Enterobacteraceae* family of *Gammaproteobacteria* are increased in faecal samples of these patients. An FMT restores the composition of the intestinal microbiota similar to the donor, dominated by *Bacteriodetes* and *Firmicutes* and a reduced amount of *Gammaproteobacteria* [173,174,175].

Little information is available on mechanisms related to how *C. difficile* suppresses specific members of the microbiota. Competitive niche exclusion is the classic mechanism that is based on ecological principles. The principle of this mechanism is the competition for limited amounts of nutrients. This mechanism can be used for the prevention or treatment of CDI, using non-toxigenic *C. difficile* (NTCD), which protects against toxigenic *C. difficile* [176]. There are also bacteria that inhibit growth or toxigenic activity by producing phages or antimicrobial peptides (bacteriocins). Bacteriocin thuricin, secreted by *Bacillus thuringensis,* has a narrow-spectrum action against *C. difficile* and an in vitro model of the faecal microbiota in the distal colon [177].

Standard treatment is a course of vancomycin or metronidazole [150]. There were two studies that compared FMT with vancomycin therapy. Due to the superior efficacy of FMT, both were stopped [178]. Studies show that donor faeces duodenal infusion for rCDI had a cure rate of 81% versus a cure rate of 31% for patients treated with the standard oral vancomycin [175].

It was concluded that an FMT should be considered for recurrent or relapsing CDI when there is failure to respond to conventional therapy. Recurrent CDI is defined as complete resolution with appropriate therapy followed by the recurrence of CDI after treatment has stopped [179]. In the case of moderate CDI, an FMT is indicated when there is no response to standard therapy for at least 1 week. For severe CDI, it is indicated when there is no response to treatment after appropriate maximal therapy for 48 h [129].

This method of treatment has also been applied with more limited success to patients with other intestinal diseases such as Crohn’s disease, ulcerative colitis, and colorectal cancer [137,180]. There is also tentative evidence that an FMT can help with non-gastrointestinal conditions such as hepatic encephalopathy, neuropsychiatric diseases, allergies, psoriasis, neurologic disorders, metabolic syndrome, and cancer that are related to intestinal dysbiosis [181].

#### An FMT in Inflammatory Bowel Disease

Inflammatory bowel disease (IBD) is a chronic inflammatory condition of the gastrointestinal tract that includes ulcerative colitis (UC) and Crohn’s disease (CD). The aetiology of these disorders is multifactorial. Genetics, changes in microbial composition, altered immune system, and environmental factors have a role in the pathogenesis of IBD. Clinical signs of IBD include diarrhoea, nausea, weight loss, loss of appetite, fever, and abdominal pain. The main aim of the management of IBD is to suppress the inflammatory response while using salicylates, corticosteroids, thiopurines, anti-tumour necrosis factor agents, and anti-integrins. There are some limitations of these types of treatments due to side effects, infections, secondary malignancies, and lack of response [182].

Physicians have found out that patients with IBD have altered faecal and mucosal bacterial microbiomes when compared to healthy controls [182]. The faecal bacterial flora of patients with IBD has been shown to differ from that of healthy individuals [183,184]. An imbalance in the microbiome has recently been considered as a possible pathologic trigger for the development of IBD [20]. Patients with IBD were found to have a general reduction in bacterial diversity with specifically reduced members of the *Bacteriodetes* and *Lachnospiraceae* phylum within the Firmicutes phylum and an increase in Proteobacteria and Actinobacteria [184,185,186]. The bacterial mucosal surface component of patients with IBD is also known to differ from that of healthy humans [187]. Bacterial invasion of the mucosa is evident in patients with CD and UC, although rarely found in healthy individuals [188,189]. In these patients, there is an increase in entero-adherent bacteria and a decrease in health-promoting bacterial communities in these patients [190].

Microbiome manipulation that restores intestinal microbiome composition has been considered a therapeutic option in the treatment of patients with IBD. A rigorous systematic review of 18 studies that include 122 patients with IBD treated with FMT found overall clinical remission rates of 36.2%. In UC patients, clinical remission was 22%; CD patients had a rate of 60.5% and in younger patients (aged 7–20 years) [191]. It appears that an FMT may be more effective for CD and in younger patients than for UC infection.

There are other studies exploring the use of an FMT for the treatment of IBD. In one study, 75 patients with active UC were included. Some were treated with an FMT and others with a water enema for 6 weeks. In patients treated with FMT, remission was 24% (defined by Mayo score <3 and complete mucosal healing), while in patients treated with a water enema, remission was only 5% [192].

It is clear that the effectiveness of an FMT in IBD is not as high as that in CDI. It is probably due to the multifactorial pathophysiology of IBD [115]. More studies are required to determine whether there is a beneficial effect in this population and to assess possible adverse outcomes. An FMT also has potential clinical applications in the treatment of a wide spectrum of conditions associated with intestinal dysbiosis. Table 7 introduces conditions associated with dysbiosis, in which the use of an FMT has potential beneficial effects.

## 6. Safety of an FMT, Alternatives, and Future Perspectives

An FMT is one of the novel methods of modulating the composition of the gut microbiota in humans. FMT is known to be the most effective therapy for recurrent CDI, but it also has a potential effect in the treatment of a wide spectrum of other conditions associated with intestinal dysbiosis. Studies have shown that FMT does not have the same dramatic impact on IBD and other diseases as it does on CDI. Although both CDI and IBD are characterized by an altered microbiome, IBD is a much more complex disease with multifaceted interactions between the host and its environment [182].

This form of bacteriotherapy is generally considered safe and well-tolerated even in high-risk patients. However, there are also opinions that, due to the unidentified composition and pathogenicity of faecal bacteria, the safety of an FMT remains controversial [201]. Most short-term risks are mild and are known to be associated with delivery methods. They include transient fevers, abdominal discomfort, diarrhoea, bloating, flatulence, elevation of inflammatory markers, and vomiting (after duodenal infusions) [191,202]. What has evoked controversies is the recent release of a safety alert from the Food and Drug Administration (FDA), warning about the potential risks of transmitting multi-drug resistant bacteria and developing subsequent life-threating infections. Several cases report infection by extended-spectrum beta-lactamase (ESBL)-producing *E. coli* after an FMT, norovirus gastroenteritis, *E. coli* bacteraemia, and cytomegalovirus infection [203,204,205,206].

In addition to the transmission of pathogens, there is also the possibility of disseminating disease-causing genes. During the process of transferring faecal material, there is some risk that some unknown components of the donor’s stool are passed onto the recipient and consequently trigger the chronic diseases (obesity, autism, cardiovascular, autoimmune, or gastrointestinal disorders) [203].

To maintain patient safety and appropriate use of an FMT, standardized protocols for donor screening, stool preparation, methods of delivery, and recipient indications for treatment are expected to emerge [148].

As mentioned above, FMT presents a risk to some patients; therefore, there are some novel alternatives to FMT known as next-generation microbiota-based therapies, which are safer than FMT. These synthetic stool products or bacterial consortia with defined microbial strains are also being developed as treatments, based on the principle of intestinal microbiota reconstitution [102,172]. The exact composition of the bacteria to be supplied is known with these stool-derived mixes, which can be regulated and replicated for future treatments. The advantage of the application of synthetic mixtures is that their application does not require the transfer of the full faeces as in an FMT. In addition, these mixtures do not include contagious pathogens and do not require donors beyond the initial isolation phase [4]. The choice of strains included in the mixture is based on replenishing the bacteria absent in the patient. In addition to safety, they are easier to manufacture and more consistent between batches [207,208].

In 2013, the use of these stool-derived mixtures was described in a study in which the mixture of 33 strains was used with a successful effect in the treatment of CDI [172]. There are other studies confirming that these synthetic mixtures of defined microbes, also called microbial ecosystem therapeutics (MET), are an available alternative to a conventional FMT for recurrent CDI [4].

In the context of alternatives to FMT, the term “personalized medicine” is being increasingly used [182]. Personalized medicine is an advanced approach that accounts for variability, including genetic, environmental, and lifestyle factors between individuals, and characterizes the unique complex disease-specific metabolic patterns of each patient [209]. This field focuses on the treatment of a specific disease considering the individual’s specific microbiome. The principle of this medicine is based on the routine analysis of the microbiome of an individual and the predictive response of an individual to different nutrients and therapeutic agents, creating the opportunity to develop new disease-specific therapeutic strategies [91].

As we mentioned above, there are some traditional methods of gut microbiome modulation, such as the administrations of probiotics, prebiotics, postbiotics, or synbiotics. Unlike FMT, which is primarily intended for the treatment of diseases, these methods are also used in the prevention of diseases; therefore, their use should be in mind [210].

However, the use of these methods is undoubtedly very effective; the effect of these approaches is highly generic and nonspecific. Traditional probiotics have been isolated from many sources such as gut and traditional fermented foods. All of them have a long history of use and have been proven to be safe. Most belong to a limited list of genera, basically, lactic acid bacteria spp. and *Bifidobacterium* spp., although there are also some members of *Bacillus* and *E. coli* for bacteria and yeast *Saccharomyces*, among others [211].

The administration of conventional probiotics promotes the improvement of intestinal barrier function, increasing IgA levels in intestinal fluids, maintaining intestinal microbiota homeostasis, and reducing pathogenic organisms in the intestinal tract through the production of antimicrobial components and the production of essential molecules [91,190]. Although probiotics have long been suggested to only affect intestinal health, current evidence suggests that they are also involved in the regulation of sleep quality, mood, and cognitive function via ‘gut- microbiota-brain axis’, a specific pathway that involves the neural, endocrine, and immune system [212,213,214,215,216,217]. Despite these qualities, the effects of conventional probiotics are very much strain-specific and also vary between hosts. Moreover, their administration aims to prevent targeted diseases rather than improve them [91]. Therefore, a method that would modulate the microbiome in a more precise personal way is needed. Because of that, the field of personalized medicine has introduced a new type of probiotic, also known as next-generation probiotics (NGPs). In fact, it is about commensal bacteria designed as probiotics, which are associated with the progression of the severity of a particular disease [218]. NGPs have been identified mainly based on a comparative analysis of the composition of the microbiota between healthy and unhealthy individuals and belong to various genera. They do not have a long history of safe use, and thus their safety is not considered to be proven [211].

Recently, the United States Food and Drug Administration (FDA) has introduced the term ‘Live Biotherapeutic Product (LBP)’, which is a biological product that contains live organisms; is applicable to the prevention, treatment or cure of a disease or condition of human beings; and is not a vaccine. This term, sometimes proposed as a substitute for NGPs, includes live biotherapeutic microorganisms and the other ingredients that make up the final LBP. This is the reason why we strongly believe that the term ‘LBP’ should not be used systematically to replace NGP [219,220].

In the future, NGPs can be used to ameliorate the target-specific disease by modulating the gut microbiota [91], for example, in the development of probiotics designed for pregnant and breastfeeding women, to prevent skin allergies, infections, and gastrointestinal problems, DM2, childhood allergies and eczema, etc. [191]. Most NGP products are not yet commercially available, and research is currently underway to select suitable candidates for target therapy.

## 7. Conclusions

Faecal microbiota transplantation represents an alternative method of therapeutic option in the case of conventional treatment failure or as a supplement to conventional therapy. In veterinary medicine, we are in the early stages of investigating the potential application of an FMT in modulating the gut microbiota. However, there is still much that we do not understand about the exact mechanism of action; it is expected that the standardization of an FMT will be established in the coming years and its indication will be expanded. As there is currently a trend in human medicine to develop the field of personalized medicine and targeted modulation of the intestinal microbiota, its further development and introduction into veterinary medicine could be a key to the treatment of many gastrointestinal and extragastrointestinal diseases in the future.

## Figures and Tables

**Table 2:** Conditions that can cause intestinal dysbiosis.

**Anatomic Abnormalities**	**Exocrine Pancreatic Insufficiency (EPI)**
◦ Blind loops ◦ Small bowel strictures ◦ Surgical resection of the ileo-colic valve ◦ Neoplasia ◦ Foreign bodies [20,32]	◦ The decreased production of pancreatic antimicrobial factors. ◦ The storage of undigested substrate in lumen leading to SIBO (small intestinal bacterial overgrowth) [20,32].
**Motility disorders**	**Chronic enteropathies**
◦ Hypothyroidism ◦ Diabetic autonomic neuropathy ◦ Scleroderma ◦ Abnormal migrating motor complexes [20,32]	◦ Intestinal inflammation maintains aerobic conditions and changes in pH in the mucosa. ◦ The reduction in the mucus layer allows the attachment of bacteria to mucosa [20,32].
**Decreased gastric acid output**	**Miscellaneous**
◦ Atrophic gastritis ◦ Administration of acid suppressing drugs (H2-blockers, omeprazole) [20,32]	◦ Decreased mucosal immunity ◦ Antibiotic induced (e.g., tylosin, metronidazole). ◦ Diets high in protein and fat and low in fiber (increase *C. perfringens* and *E. coli*) [20,32].

**Table 3:** Consequences of gut dysbiosis.

Types of Dysbiosis	Consequences
Storage of an abnormal substrate in the intestinal lumen (undigested nutrients, medications) [32]. Disruption of proper microbiome function caused by lack of commensal bacteria (*C. hiranonis*) [47]. Increase in the total number of bacteria, primarily in the small intestine [32]. Increased mucosa- adherent bacteria [32].	Increase in bacterial species, causing osmotic/secretory diarrhoea (conversion of fatty acids to hydroxystearic acids, metabolites of mycophenolate motefil) [32]. Bacterial overgrowth (*C. difficile, C. perfringens, E. coli*) caused by lack of conversion from primary to secondary BAs. Lack of anti-inflammatory microbial-derived metabolites [63]. Increased production of microbial metabolites leading to osmotic/secretory diarrhoea. Activation of inflammatory reactions [32]. Increased adhesion of bacteria to the intestinal mucosa causes increased inflammatory reactions [32].

**Table 4:** Reference intervals of abundances of 7 bacterial groups and final DI.

	Normal Abundance	Changes Seen in Dogs with Dysbiosis
*Faecalibacterium*	3.4–8.0	decreased
*Turicibacter*	4.6–8.1	decreased
*Streptococcus*	1.9–8.0	increased
*E. coli*	0.9–8.0	increased
*Blautia*	9.5–11.0	decreased
*Fusobacterium*	7.0–10.3	decreased
*C. hiranonis*	5.1–7.1	decreased
Dysbiosis index	<0 normal 0–2 equivocal >2 dysbiosis	[42,64]

Note: Data expressed logDNA/gram of faeces.

**Table 5:** Types of modulation of the gut microbiota.

Type of Modulation	Mechanism	Side Effects
**Diet**	A highly digestible diet reduces the storage of undigestible substrate in the intestinal lumen [72,73].	Only in the case of food hypersensitivity or difficult to digest food [32].
**Prebiotics**	Production of SCFAs for the growth of beneficial bacteria, binding of deleterious bacterial metabolites (e.g., psyllium has BA- binding properties) [39,70].	Sometimes flatulence, diarrhoea [32].
**Probiotics**	Improvement of barrier function, immunomodulatory and antimicrobial effect [74].	Rare [32].
**Synbiotics**	Products that contain probiotics and prebiotics.	
**Antibiotics**	Reduction of total bacterial load, suppression of immune stimulation, and conversion of toxic metabolites [66,67].	- Long-term changes in the microbiota composition. - Risk of antimicrobial resistance [32].
**Postbiotics**	Immunomodulatory, anti-inflammatory, antioxidant, and anticancer effects [30].	Rare [30].
**FMT**	Reconstruction microbial composition and some microbial-derived metabolites [32].	Diarrhoea, flatulence, bloating, fever, vomiting [2,3].

**Table 6:** Recommended selection criteria for canine faecal donors.

**History and Physical Examination**
Age between 1 and 10 years; No travel history outside the local area; No history of chronic GI disease, cancer, allergies, or autoimmune diseases; Healthy state in the last 6–12 months; No antibiotics in the last 12 months; Optimal weight (not overweight or underweight); Fed a balanced diet; Normal faecal consistency; Feeding canine donors with a hydrolyzed diet for several weeks before and during collection is recommended [153,160].
**Laboratory screening**
No significant changes in the hematology and biochemistry profile; Normal value of pancreatic enzymes, pancreatic immunoreactivity, and trypsin-like immunoreactivity); Optimal serum concentration of cobalamin and folate (= tests of intestinal functions); No presence of endocrinopathy (serum cortisol, thyroxine, TSH concentrations); Negative for faecal parasites; Negative for faecal pathogens (*Salmonella* spp., *Campylobacter* spp., etc.) [153,160].
**Evaluation of the faecal microbiota**
Faecal dysbiosis index less than 0 [42].

**Table 7:** Conditions in which an FMT has potential effects.

**Metabolic Diseases**	**Autoimmune Diseases**
Obesity Diabetes mellitus Metabolic syndrome NAFLD [193,194] Cardiovascular diseases [48]	Rheumatoid arthritis Idiopathic thrombocytopenic purpura Sjögren’s syndrome Systemic lupus erythematosus Hashimoto *’s* thyroiditis [195]
**Neuropsychiatric disorders**	**Allergic disorders**
Parkinson’s disease Multiple sclerosis Autism Myoclonic dystonia Chronic fatigue syndrome [48]	Atopy Food allergy Asthma [48]
**Cancer**	**Functional gastrointestinal disorders**
Gastrointestinal cancer Gastric cancer Colorectal cancer Hepatocellular carcinoma Pancreatic cancer [196,197,198,199] Extragastrointestinal cancer Breast cancer Melanoma Prostate cancer Lymphoma	Irritable bowel syndrome [200]

## Data Availability

Not applicable.
